# Flat-Field Super-Resolution Localization Microscopy with a Low-Cost Refractive Beam-Shaping Element

**DOI:** 10.1038/s41598-018-24052-4

**Published:** 2018-04-04

**Authors:** Christopher J. Rowlands, Florian Ströhl, Pedro P. Vallejo Ramirez, Katharina M. Scherer, Clemens F. Kaminski

**Affiliations:** 1 Department of Chemical Engineering and Biotechnology, University of Cambridge, Cambridge, UK; 2 Department of Bioengineering, Imperial College London, South Kensington, UK

## Abstract

Super-resolution single-molecule localization microscopy, often referred to as PALM/STORM, works by ensuring that fewer than one fluorophore in a diffraction-limited volume is emitting at any one time, allowing the observer to infer that the emitter is located at the center of the point-spread function. This requires careful control over the incident light intensity in order to control the rate at which fluorophores are switched on; if too many fluorophores are activated, their point-spread functions overlap, which impedes efficient localization. If too few are activated, the imaging time is impractically long. There is therefore considerable recent interest in constructing so-called ‘top-hat’ illumination profiles that provide a uniform illumination over the whole field of view. We present the use of a single commercially-available low-cost refractive beamshaping element that can be retrofitted to almost any existing microscope; the illumination profile created by this element demonstrates a marked improvement in the power efficiency of dSTORM microscopy, as well as a significant reduction in the propensity for reconstruction artifacts, compared to conventional Gaussian illumination.

## Introduction

Over the last decade, super-resolution microscopy has become a routine technique for imaging features below the diffraction limit of light, and one of the most commonly used methods is single-molecule localization microscopy, often referred to as PALM^1^ or STORM^2^, or the closely-related techniques of GSDIM^3^ or *d*STORM^4^. In these techniques, all fluorophores in a sample are driven into, or start in, a long-lived dark state. A small fraction of fluorophores is allowed to stochastically return to a bright state, either by photoactivation using short-wavelength light (PALM or STORM) or spontaneously (GSDIM or *d*STORM, although photoexcitation can also be used in these techniques as well); the precise photophysical details of these transitions have been reviewed elsewhere^5^. Upon conventional fluorescence excitation with a high-intensity laser, these active fluorophores are rapidly imaged, and then either bleached, or driven back to the dark state, so that no two fluorophores are active in the same diffraction-limited volume simultaneously. The location of each fluorophore can be determined by fitting the resulting point-spread function^6^, and this process is repeated multiple times in order to build up a super-resolved image.

Because the rate at which fluorophores transition between the dark and bright states is crucial to the success of the technique, controlling the illumination intensity is very important. In the case of PALM and STORM, the rate of on-switching is controlled by the intensity of activation light striking the sample. A non-uniform excitation would therefore lead to bias and possibly errors in the resulting image, as the activation rate non-uniformity means that the image will have different numbers of localization events in different regions. The rate at which multiple fluorophores are activated in the same diffraction-limited volume may also vary across the image and hence some regions will ‘waste’ their fluorophores by having an unacceptably high rate of overlaps. For GSDIM and *d*STORM, the rate of on-switching is a combination both of the fluorophores’ spontaneous return to the ground state (the rate of which is controlled by the contents of the imaging buffer solution) as well as an optional ultraviolet light that increases the rate at which the fluorophores return to the ground state. This complex interplay of different photophysical phenomena may also lead to sub-optimal switch-on and imaging rates in regions that are not illuminated with an optimal intensity.

**Figure 1:**
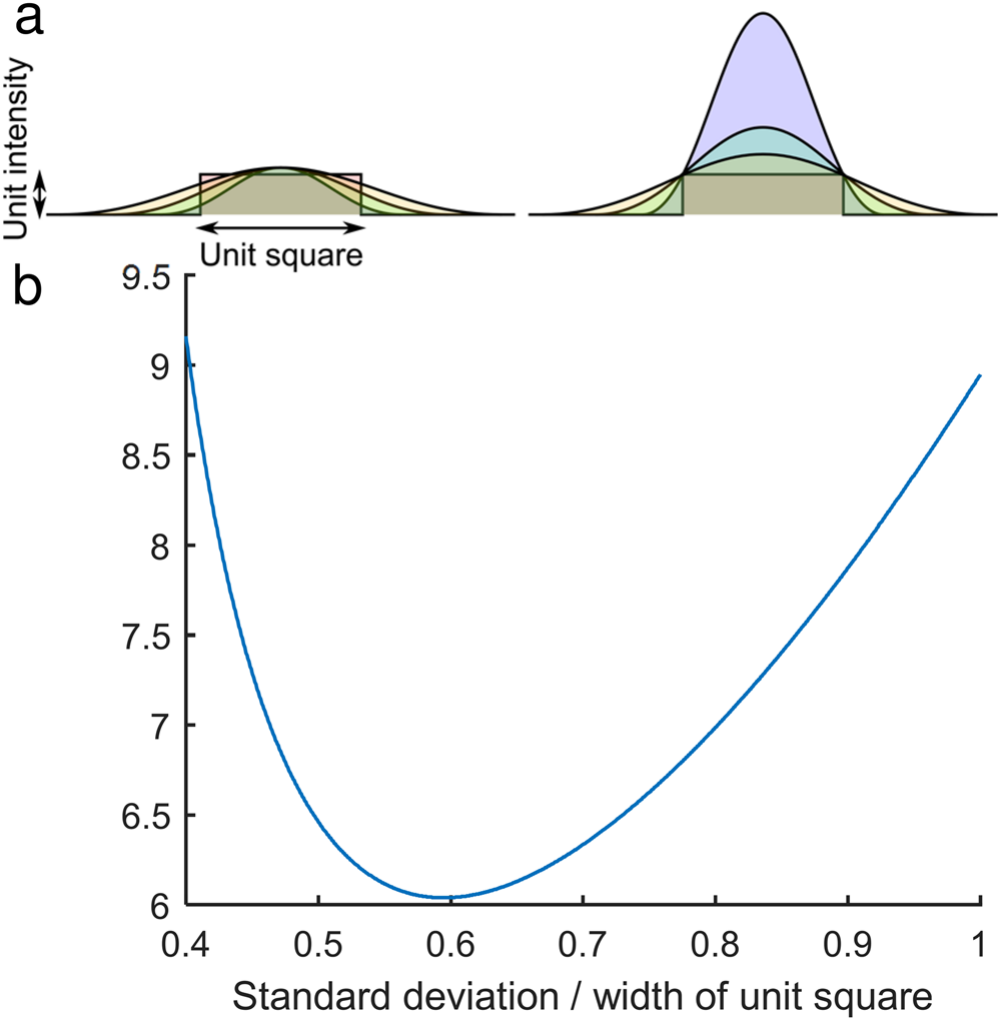
Power efficiency of a Gaussian beam versus a top-hat beam. (**a**) Gaussian beams with different standard deviations (left) must be scaled by different amounts to make sure that all pixels in the illuminated area are above threshold (right). (**b**) The result is wasted power, both at the edges, and towards the center of the illuminated area. This waste is quantified by plotting the area of the Gaussian profile, since the area of the top-hat is unity. The calculation can be performed both in 1D and 2D; results are provided for the 2D case.

In conventional instruments, the illumination profile is Gaussian, therefore there is a tradeoff between field flatness and photon efficiency (defined as the number of photons in the illuminated area as a fraction of those produced by the laser); one can use just the very center of the Gaussian beam, but this wastes all the power on the edges. Alternatively, one can use more of the beam, at the cost of under-exposing the edges. Figure 1 illustrates this efficiency tradeoff; it represents a numerical simulation of the increase in the power required to illuminate every point within a unit square above a threshold value (taken to be unity). The normalized standard deviation of the Gaussian profile is plotted along the X axis, and the amplitude required to illuminate all the pixels above threshold is then calculated. Once the amplitude and standard deviation are known, the total volume of the Gaussian profile can then be plotted, and since the volume of the top-hat profile is unity, this also plots the increase in required power over that of a top-hat profile. The optimum power efficiency (i.e. where this wasted illumination is minimized) was determined to be ~6.04× greater than that for a 2D top-hat beam profile. This optimum occurs when the width corresponding to one standard deviation of the intensity profile of the Gaussian beam measures approximately 0.6 times the length of the side of the illuminated square.

A further, more subtle, concern with the use of Gaussian illumination arises due to the assumption that a super-resolved image is a faithful representation of a wide-field image; with a sufficiently large number of localized points, the image should look just like a high-resolution version of the corresponding confocal microscope image. In order for this assumption to be true, the probability that a fluorophore is activated and successfully localized should be uniform across the image; if it is not, the apparent fluorophore density will not accurately reflect the actual density of fluorophores and the resulting image will be ‘biased’ towards regions where the illumination intensity is optimal, affecting both the apparent resolution as well as the apparent brightness.

These limitations have led to interest in techniques for ensuring a uniform illumination profile, many of which involved time-averaging a speckle pattern, such as through the use of shaken multimode fibers^7^, vibrating membranes^8^ or spinning diffusers^9^. The fact that these approaches introduce moving components to the optical table means that extreme care must be taken to avoid vibration of the sample, but in addition, they make inefficient use of laser power, both coupling light into modes that do not pass into the microscope aperture, as well as in some cases, forming a circle when the camera sensor is a square (or rectangle). Time-averaging can also place constraints on the speed at which images can be taken, since the speckle needs to be averaged faster than the switch-on rate of the fluorophores, which in turn limits the maximum achievable frame-rate. More recent approaches for generating a uniform, or ‘top-hat’, illumination profile include the use of the well-known^10^ beam homogenizer microlens configuration^11^ to couple the laser into the desired profile more efficiently. Other methods include the use of diffractive beamshapers^12,13^, or even simple image projection, but in this paper we present the use of a commercially-available, low-cost, refractive beamshaper for generating a top-hat illumination profile at the sample plane of a PALM/STORM microscope with high efficiency. We dub this technique Flat Intensity Distribution Optimization, or FIDO. This device can be retrofitted to essentially any PALM/STORM microscope, and with suitable magnification and optional cleanup of the edges, produces an exceptionally flat field suitable for many types of localization microscopy. Unlike a beam homogenizer, there is only a single element to align, and no requirement for a rotating diffuser to overcome speckle artifacts, although in cases where, for example, tissue scattering introduces unwanted laser speckle, a rotating diffuser can be placed at the top-hat plane. Furthermore, unlike diffractive solutions, the refractive element works for a wide range of wavelengths.

## Materials and Methods

### *d*STORM Microscope

**Figure 2:**
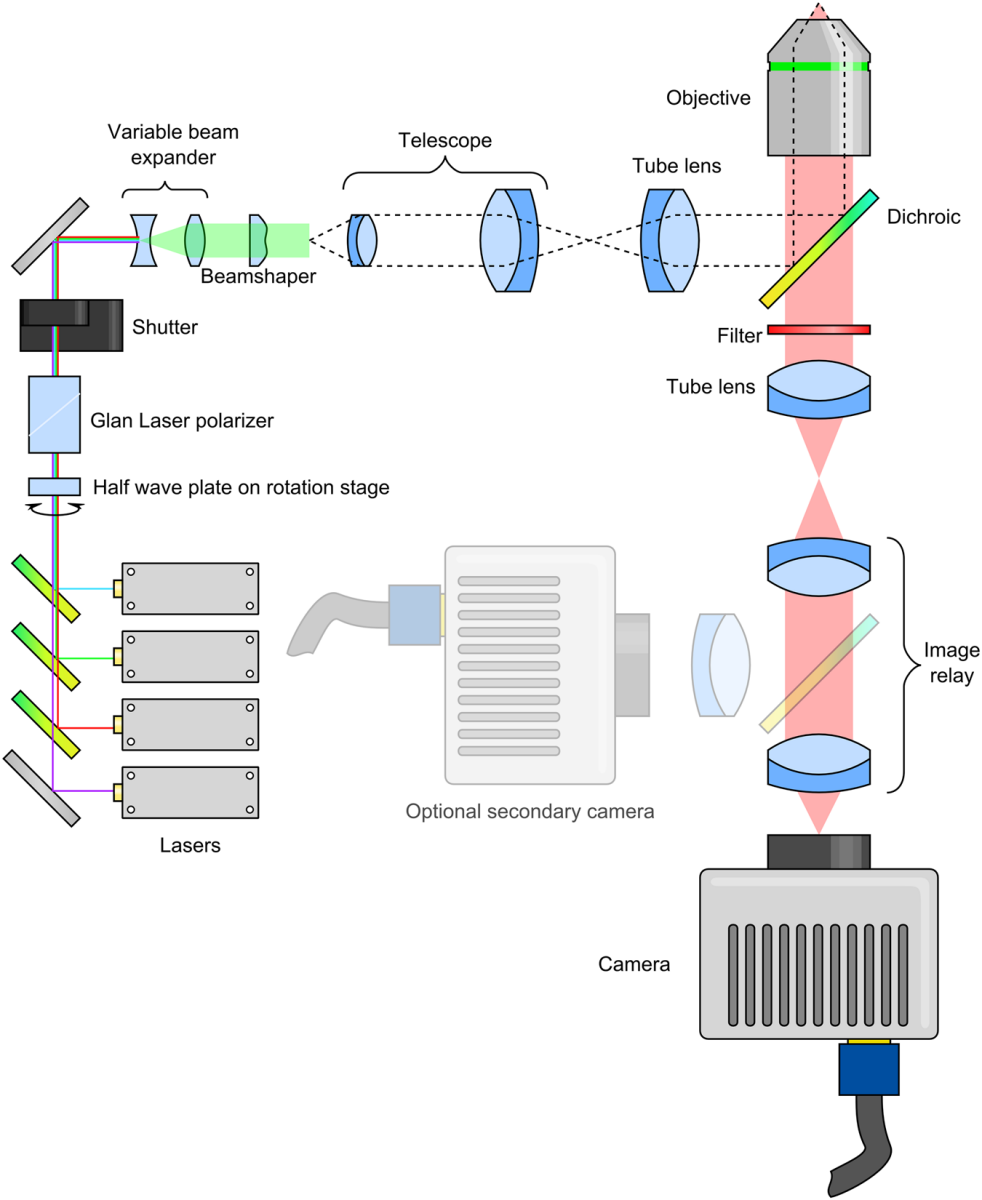
Optical layout. Four lasers are combined using dichroic mirrors and expanded to ~5 mm diameter. The beams then pass into a beamshaper, which produces a top-hat profile. This is then imaged onto the sample via a telescope and reflection off a dichroic mirror. Fluorescent light from the sample then passes through the dichroic mirror before being filtered to remove any excitation light, and imaged onto an EMCCD camera through a 1.3× image relay.

The *d*STORM microscope was based on an Olympus IX73 frame, and the layout is illustrated in Fig. 2. Four excitation lasers, at wavelengths of 488 nm (Coherent Sapphire 488-300 CW CDRH), 532 nm (Cobolt Samba 500 532 nm), 561 nm (Cobolt Jive 500 561 nm) and 647 nm (MPB Communications Inc. VFL-P-300-647-OEM1-B1) were combined using dichroic mirrors (Chroma ZT502RDC, ZT532RDC and ZT568RDC) onto a common optical path. Each laser had a home-built shutter based on a stepper motor (Wantal Stepper Motor 42BYGHW2058) controlled by an Arduino A000066. The dynamic range of power control was extended by passing all four beams through a half wave plate (Thor Labs AHWP05M-600) and polarizer (Thor Labs GL10-A). The half wave plate could be rotated automatically using a motor-driven rotation stage (Thor Labs PRM1/MZ8). Finally a shutter (Thor Labs SH05/M) was used to block the beam path for exposure control of all beams simultaneously.

The combined beams were routed into a separate STORM module, where they were expanded to approximately 5 mm 1/e^2^ diameter using an Edmund Optics 87–569 variable beam expander, before passing through the top-hat beamshaper (Topag GTH-5-250-4-VIS). The 4 mm × 4 mm square illumination produced by the beamshaper then was trimmed to the correct size to fill the camera by an adjustable rectangular aperture (Owis SP 60) before being magnified by a 3.3× telescope consisting of two doublet lenses (Thor Labs AC254-075-A-ML and AC508-250-A-ML). The image was then relayed via a periscope to the image plane of the 400 mm focal length tube lens (Thor Labs AC508-400-A-ML). Images of this plane can be seen in Supplementary Figure 1.

After the light passed into the microscope frame from the tube lens, it was reflected from a dichroic mirror (Chroma ZT488/561/647rpc) through the 100×, 1.49 numerical aperture objective lens (Olympus UAPON100XOTIRF) to the sample. For super-resolution experiments, the sample was disengaged from the microscope frame by a nosepiece stage (Olympus IX2-NPS), in order to minimize vibration.

Light from the sample passed through the dichroic and through a 25 mm diameter optical filter (Semrock FF01-680/42-25), to the microscope side port. The image was relayed onto the camera (Andor iXon Ultra 897) by a 1.3× magnification Cairn Twincam image splitter, but the second port of the image splitter was not utilized during these experiments. The resulting size of the camera pixels on the sample was measured to be 118 nm. A diagram of the optical layout can be seen in Fig. 2.

### Microtubulin-labelled fibroblasts

Immunostaining was performed in human foreskin fibroblast (HFF) cells (American Type Culture Collection) cultured with Dulbecco’s Modified Eagle’s Medium (DMEM, high glucose) supplemented with 10% fetal bovine serum, 1% GlutaMAX, penicillin and streptomycin, and incubated at 37 °C with 5% CO_2_. Cells were plated in a LabTek 8-well coverglass chamber at ~30,000 cells per well 12 hours prior to fixation. The immunostaining procedure for microtubules consisted of: fixation for 10 min with 3% paraformaldehyde and 0.1% glutaraldehyde in phosphate-buffered saline (PBS); washing with PBS; blocking and permeabilization for 20 min in PBS containing 3% bovine serum albumin and 0.25% (v/v) Triton X-100 (blocking buffer (BB)); staining for 40 min with primary antibody (rabbit anti-tubulin (ab18207, Abcam)) diluted in BB to a concentration of 10 µg/mL; washing with PBS containing 0.2% bovine serum albumin and 0.1% (v/v) Triton X-100 (washing buffer, WB); incubation for 30 min with secondary antibody (Alexa Fluor 647-labelled donkey anti-rabbit (ab150067, Abcam)) at a concentration of 1 µg/mL in BB; washing with WB and subsequently with PBS; postfixation for 10 min with 3% paraformaldehyde and 0.1% glutaraldehyde in PBS; and finally washing with PBS.

*d*STORM imaging of immunostained cells was performed in an imaging buffer solution. The buffer solution was added just before image acquisition and was prepared following the protocol by van de Linde^14^. In particular, a 50 mM solution of mercaptoethylamine in PBS was used, which is optimal for the employed Alexa Fluor 647 label. No additional oxygen scavenging system was necessary.

### Quantum dot test slides

10 µl of a suspension of Quantum dots in water (carboxylic acid functionalized, 665 nm emission, Sigma Aldrich 900227) was placed on a coverslip and allowed to dry. The coverslip was then sealed to a microscope slide using Fluoromount G (Sigma Aldrich). The edges of the dried spot, which exhibited the ‘coffee ring effect’ were found to produce the most uniform fluorescence, and were used wherever possible.

### Super-resolution imaging

Super-resolution images were taken using the *d*STORM microscope described previously. Microtubulin-labelled fibroblasts in *d*STORM buffer were placed on the microscope and a suitable cell located by low-intensity fluorescence microscopy at 647 nm excitation wavelength. Once the cell had been located, the sample was isolated from the microscope frame using the nosepiece stage, and a wide-field fluorescence image taken. The laser was then increased to maximum power, and once blinking behaviour was confirmed, 12000 frames were taken using the Andor Solis camera control software. Once imaging was complete, the beamshaper was either added to, or removed from, the optical path, and the imaging process repeated.

Fluorophore localization was performed using rapidSTORM^15^; the super-resolved image was generated using rapidSTORM itself, while further analysis of the fluorophore density was performed by importing the fluorophore locations into Matlab 2017a and performing the process using custom-written scripts.

### Data availability

Raw data from the super-resolution experiments is available from Zenodo, 10.5281/zenodo.1095007.

## Results and Discussion

An existing super-resolution microscope was retrofitted with the refractive beamshaper, the layout of which can be seen in Fig. 2. Briefly, four lasers are combined, the beam expanded to 5 mm diameter before passing into the top-hat beamshaper. The top-hat image then was magnified using a telescope, before being imaged onto the sample using a microscope.

### Incorporation and alignment

Retrofitting a microscope to include the beamshaper is straightforward; a variable beam expander is used to ensure that the beam is close to 5 mm 1/e^2^ diameter, and once this is achieved, simply placing the beamshaper into the beam is sufficient to produce a crude top-hat shape at a distance 250 mm from the beamshaper’s surface. Alignment can be improved by observing the top-hat and centering the beam to maximize uniformity by moving the incident beam in the direction of the darker side of the image. Finally, the beam diameter can be optimized by returning to the beam expander and changing the beam diameter while observing the edges of the top-hat. If the edges are bright, relative to the rest of the field, the beam is too large. If the edges are smooth and ‘blurry’, the beam is too small.

Finally, once a suitable top hat is produced, it is expanded using a simple telescope to match the field of view of the camera. For fine tuning, the top-hat may be optionally ‘trimmed’ using a pair of adjustable slits so as not to illuminate regions outside of the camera’s field of view. This is especially important for a camera such as the iXon Ultra 897 EMCCD used in this work; in this and similar cameras, a crop mode is available for increased acquisition speeds, but to avoid image artifacts there must be no exposure of the pixels outside of the cropped area.

### Illumination uniformity

**Figure 3:**
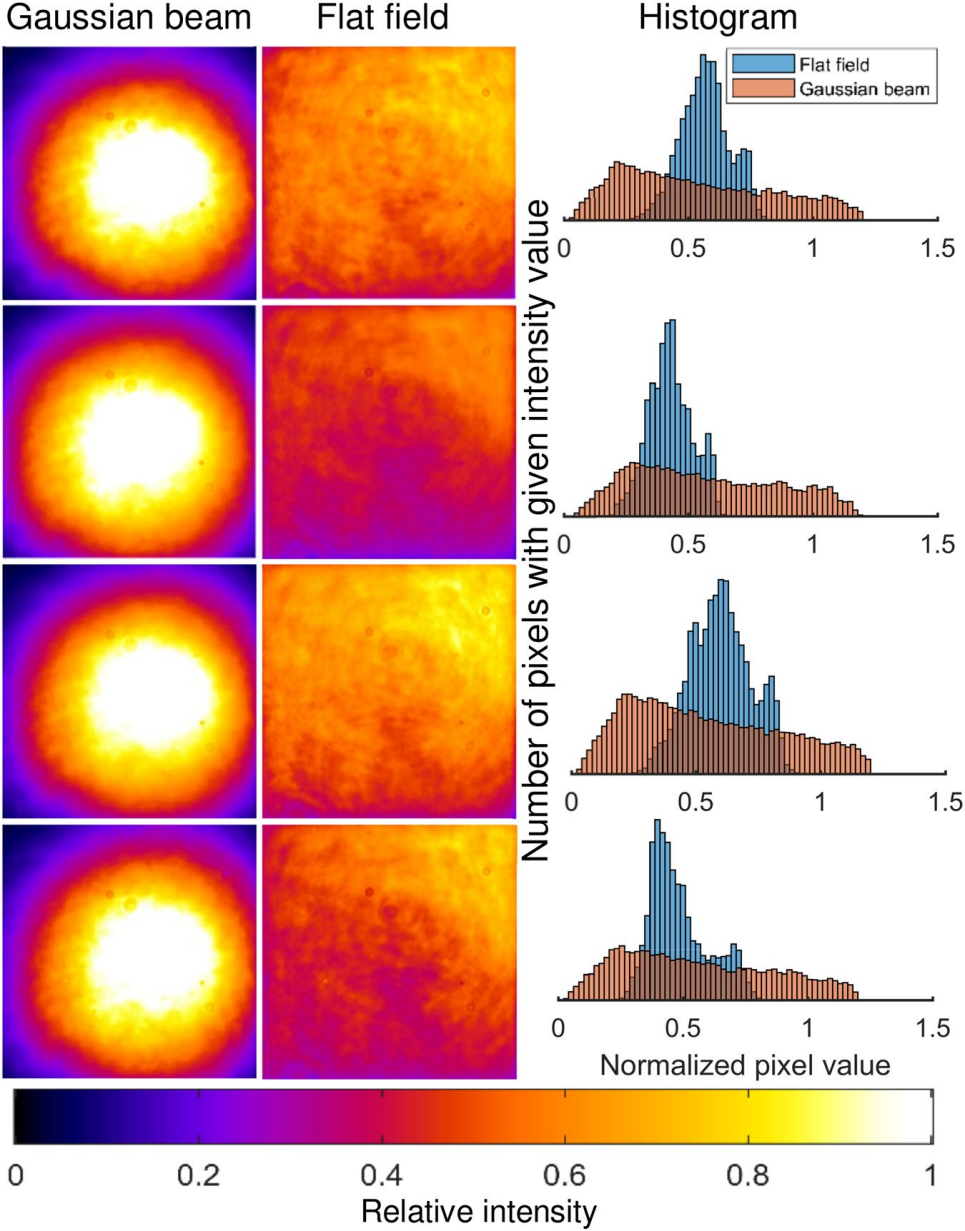
Illustrating the improvement in pixel uniformity of the top-hat beamshaper versus a Gaussian beam. The excitation wavelength was 488 nm. Four different measurements were taken at different locations on a sample consisting of a dried layer of quantum dots. The field of view is 60.4 µm × 60.4 µm for all images, and the colour map is illustrated at the bottom of the figure. Display ranges are identical for each pair of images; the lower limit is the dark level, and the upper limit is 1.2× the 95^th^ percentile value for each image.

Illumination uniformity was assessed using a dried layer of quantum dots, since quantum dots are bright, do not appreciably bleach, and have an extremely broad absorption spectrum stretching well into the ultraviolet. Images of the resulting fluorescence emission can be seen in Fig. 3, along with histograms illustrating the improved uniformity afforded by the top-hat beamshaper relative to the Gaussian beam. Power efficiency was excellent; the intensity of the top-hat beam was 98.3% of that of the Gaussian beam, which was considered a negligible loss. Even once the beam had been ‘trimmed’ to fit the camera sensor, the power within the top-hat profile was 90.6% that of the Gaussian beam.

### Wavelength sensitivity

**Figure 4:**
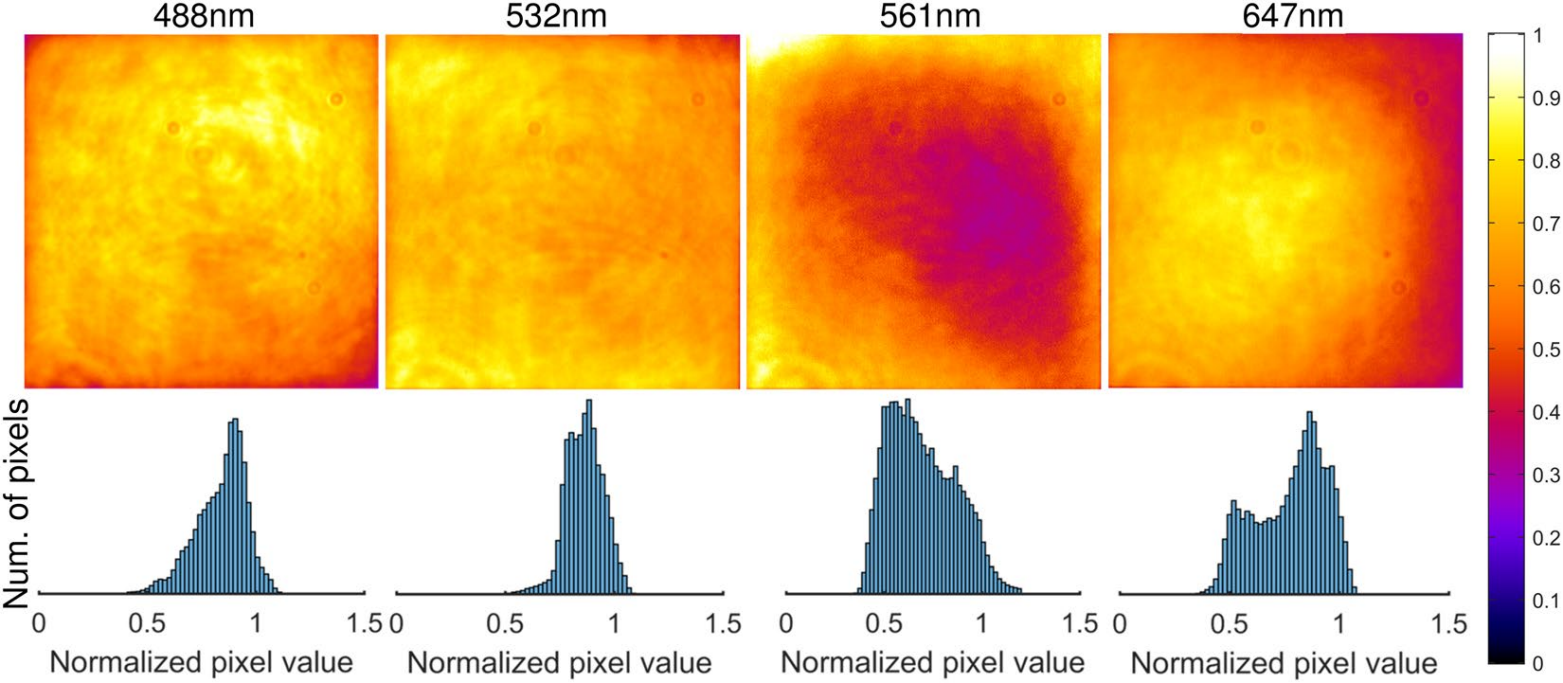
Utilizing the beamshaper with different wavelengths (488 nm, 532 nm, 561 nm and 647 nm). Images are scaled from the dark level to 1.2× the 95^th^ percentile value, and the full colour scale is indicated. Histograms are normalized to the same range.

Operation of the beamshaper was confirmed using four different lasers spanning the visible spectrum; 488 nm, 532 nm, 561 nm and 647 nm. In all cases, the test sample consisted of the same quantum dots employed previously, since their excitation spectrum spanned almost the entire visible spectrum. Despite the fact that each laser had a slightly different beam diameter, and consequently did not optimally match the input requirements of the beamshaper, the results (see Fig. 4) demonstrate that the wavelength sensitivity was acceptably low; the top-hat shape was clearly preserved regardless of which laser was used. If increased uniformity is desired, a variable beam expander can be employed for each laser, but this was deemed too costly for the marginal improvement in uniformity it was likely to bring. In our case, the 561 nm beam appeared to be slightly too large, as evidenced by the drop in intensity at the center of the image, but the other wavelengths appeared consistent with the results for 488 nm.

### Super-resolution imaging results

**Figure 5:**
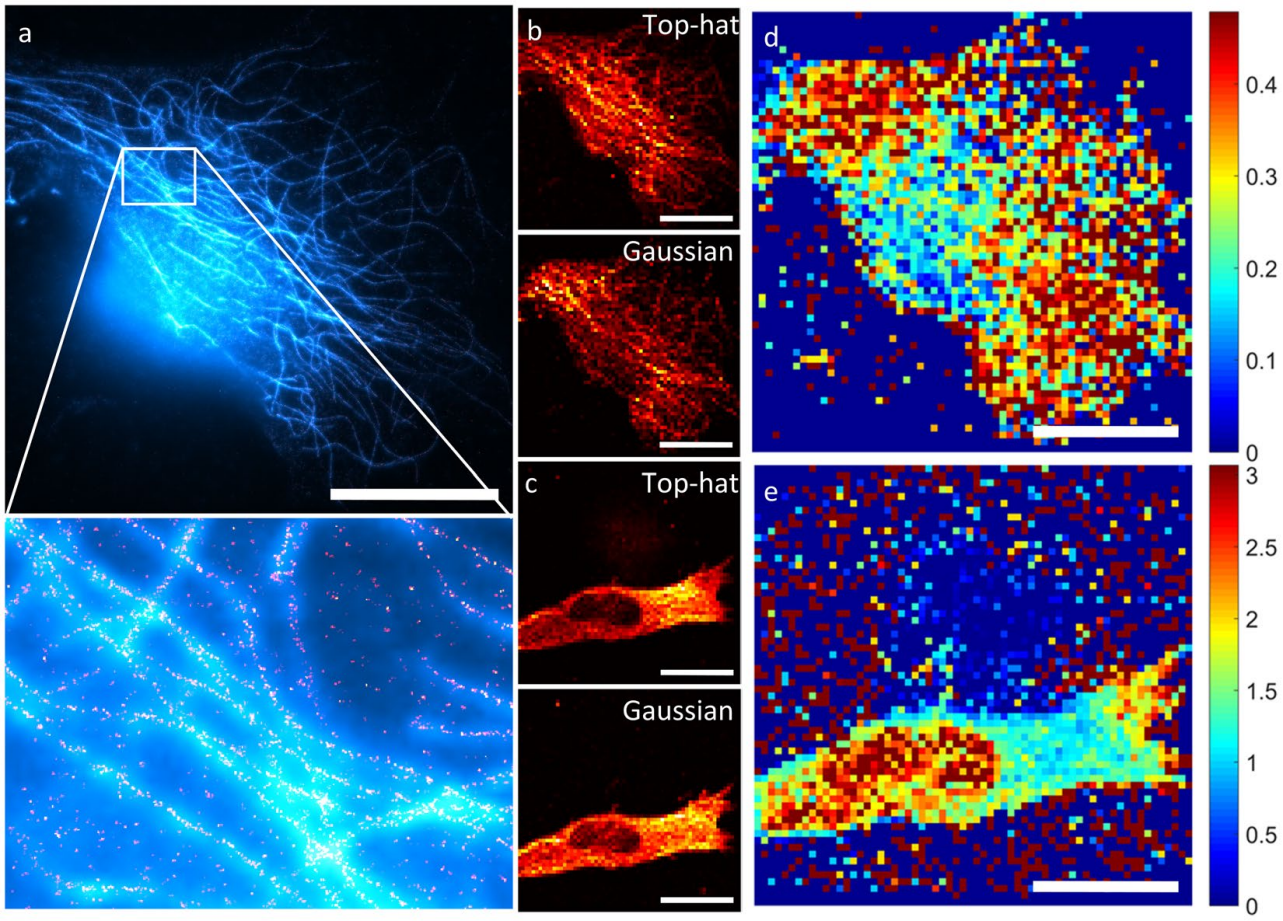
Comparing dSTORM imaging with top-hat and Gaussian beam profiles. All scale bars are 20 µm. (**a**) An example of a dSTORM image taken using a top-hat illumination profile. The cyan channel corresponds to the wide-field image taken before the super-resolution imaging process, and the red/yellow channel is the reconstructed super-resolution image. (**b**) A map of the number of identified fluorophores in (**a**) as a function of position in the image, for top hat illumination (top) and Gaussian illumination (bottom). Note that this is not a super-resolution image. (**c**) Ratio of the Gaussian fluorophore distribution over the top-hat fluorophore distribution. The ratio lies predominantly below unity because the top-hat illumination was performed first, so there are fewer identified fluorophores for the Gaussian illumination due to photobleaching. (**d**) A map of the number of identified fluorophores as a function of position in a different image, for top hat illumination (top) and Gaussian illumination (bottom). This time the Gaussian illumination was performed first. (**e**) Ratio of the Gaussian fluorophore distribution over the top-hat fluorophore distribution. The reversed order of the illumination profiles means that the ratio now lies predominantly above unity.

To demonstrate the performance advantage of flat-field illumination, four samples were illuminated with both the top-hat and the Gaussian beams, with the order of the illumination being reversed for one half of the samples so as to avoid the possibility that the results were caused by photobleaching. The results, two cells of which are shown in Fig. 5, demonstrate that the top-hat beamshaper can be used to perform localization microscopy over large areas.

To investigate the effect of illumination uniformity on the reconstructed images, several microtubulin-labelled fibroblasts were imaged with both Gaussian and top-hat illumination profiles. The density of identified fluorophores was then calculated by constructing a low-resolution (64 × 64) ‘map’ of the image, and summing all the fluorophores identified within each pixel (see Fig. 5b,d). Fluorophores which persisted across frames (defined as being located within 100 nm of an identified fluorophore in the frame immediately preceding it) were removed from the dataset. If the Gaussian profile has no effect on the likelihood that a fluorophore is successfully localized, then dividing one distribution by the other should result in a uniform value throughout the image (subject to large amounts of noise in regions with few fluorophores). What is actually observed is that there is a pronounced reduction in the number of observed fluorophores in the center of the image, where the Gaussian beam is most intense, relative to the edges. In these regions, images taken using Gaussian illumination under-count the number of fluorophores by a factor of approximately 3, which is both non-intuitive and a significant impediment to quantitative analysis of PALM/STORM images. Thus not only is the top-hat profile more power efficient than the Gaussian profile, it imparts minimal bias into the reconstructed images.

## Conclusions

In conclusion, we have presented FIDO, a simple, low-cost modification to essentially any PALM/STORM microscope, able to operate over the entire visible spectrum and with minimal requirements in terms of alignment. The theoretical power efficiency is over six times better than for a Gaussian beam, and even with the use of a pair of orthogonal slits to clean up the beam, the power throughput was more than 90% of that of a Gaussian beam.

Furthermore, the uniformity serves to reduce a pronounced bias in the reconstruction of super-resolved images, since the density of localizations was shown to be strongly dependent on the incident intensity.

## Electronic supplementary material

Supplementary Information

Illustration of camera artefacts
