# Supplementary material for: Flat-Field Super-Resolution Localization Microscopy with a Low-Cost Refractive Beam-Shaping Element
Source: Sci Rep. 2018 Apr 4;8:5630. doi: 10.1038/s41598-018-24052-4 (PMC5884788; doi:10.1038/s41598-018-24052-4)
Supplement: Supplementary file 1 — Supplementary Information [file 41598_2018_24052_MOESM1_ESM.pdf]

Flat-Field Super-Resolution Localization Microscopy with a Low-Cost Refractive Beam-Shaping Element

Christopher J Rowlands[a,b*†], Florian Ströhl[a†], Pedro P Vallejo Ramirez[a], Katharina M Scherer[a] and Clemens F Kaminski[a]

[a]*Department of Chemical Engineering and Biotechnology, University of Cambridge, Cambridge (UK)*

[b]*Department of Bioengineering, Imperial College London, South Kensington (UK)*

[†]These authors contributed equally to this work

*Corresponding author: c.rowlands@imperial.ac.uk, Tel: +44 1223 762924, Fax: +44 1223 334796

Supplementary Video 1:

An illustration allowing readers to distinguish the intensity of the projected pattern from the camera imaging artefacts. The experimental configuration consists of a camera on a rotation mount, which is slowly rotated while a top-hat beam is projected on the image sensor. Artefacts due to the camera should not appear to rotate.

Intermediate plane images

Images of the laser excitation 250mm after the beamshaper (and before the telescope) can be seen in Supplementary Figure 1. The images were taken by first reducing the laser intensity using a neutral density filter, before placing a camera (Point Grey BFS-U3-51S5M-C) at the required location. The superimposed hexagonal interference pattern and 'dust specks' are camera artefacts, which can be shown by rotating the camera about the optical axis (see Supplementary Video 1): camera artefacts are stationary while the underlying pattern rotates.

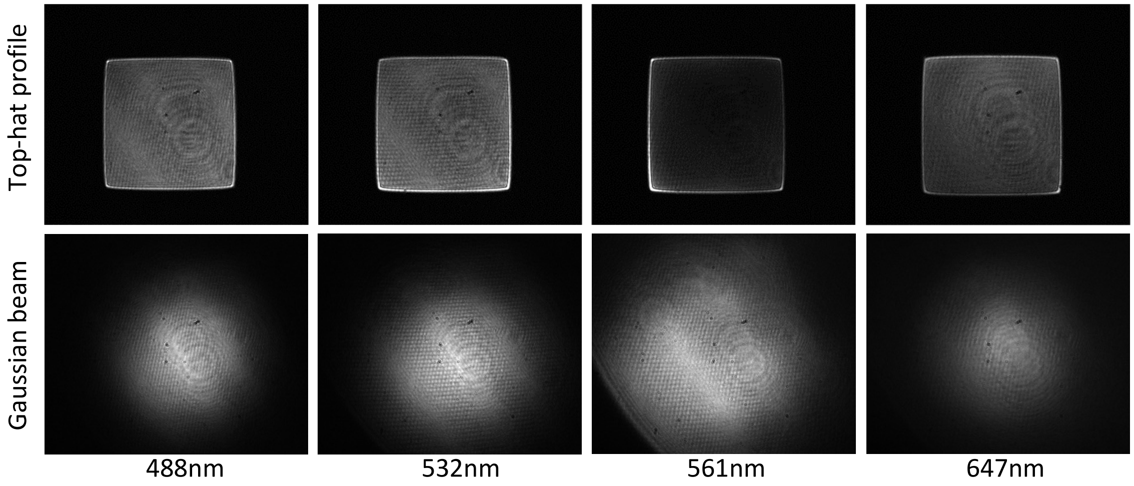

Supplementary Figure 1: Imaging the laser intensity at the intermediate focal plane, for different wavelengths. An image of the equivalent Gaussian beam is also provided for comparison; this was achieved by simply removing the beamshaper from the beam path.
